# Efficacy and safety of flexible versus rigid endoscopic third ventriculostomy in pediatric and adult populations: a systematic review and meta-analysis

**DOI:** 10.1007/s10143-021-01590-6

**Published:** 2021-06-25

**Authors:** Alessandro Boaro, Bhargavi Mahadik, Anthony Petrillo, Francesca Siddi, Sharmila Devi, Shreya Chawla, Abdullah M. Abunimer, Alberto Feletti, Alessandro Fiorindi, Pierluigi Longatti, Francesco Sala, Timothy R. Smith, Rania A. Mekary

**Affiliations:** 1 Computational Neurosciences Outcomes Center, Brigham and Women’s Hospital, Harvard Medical School, Boston, MA USA; 2 Institute of Neurosurgery, Department of Neurosciences, Biomedicine and Movement Sciences, University of Verona, Verona, Italy; 3 School of Pharmacy, Massachusetts College of Pharmacy and Health Sciences University, Boston, MA USA; 4 Faculty of Life Sciences and Medicine, King’s College London, London, UK; 5 Department of Neurosurgery, University of Brescia, Brescia, Italy; 6 Department of Neurosurgery, University of Padova, Padova, Italy

**Keywords:** Flexible neuroendoscopy, Rigid neuroendoscopy, Endoscopic third ventriculostomy, Hydrocephalus, Efficacy, Complications

## Abstract

Endoscopic third ventriculostomy (ETV) is a well-established surgical procedure for hydrocephalus treatment, but there is sparse evidence on the optimal choice between flexible and rigid approaches. A meta-analysis was conducted to compare efficacy and safety profiles of both techniques in pediatrics and adults. A comprehensive search was conducted on PubMED, EMBASE, and Cochrane until 11/10/2019. Efficacy was evaluated comparing incidence of ETV failure, while safety was defined by the incidence of perioperative complications, intraoperative bleedings, and deaths. Random-effects models were used to pool the incidence. Out of 1365 studies, 46 case series were meta-analyzed, yielding 821 patients who underwent flexible ETV and 2918 who underwent rigid ETV, with an age range of [5 days–87 years]. Although flexible ETV had a higher incidence of failure in adults (flexible: 54%, 95%CI: 22–82% vs rigid: 20%, 95%CI: 22–82%) possibly due to confounding due to etiology in adults treated with flexible, a smaller difference was seen in pediatrics (flexible: 36%, pediatric: 32%). Safety profiles were acceptable for both techniques, with a certain degree of variability for complications (flexible 2%, rigid 18%) and death (flexible 1%, rigid 3%) in pediatrics as well as complications (rigid 9%, flexible 13%), death (flexible 4%, rigid 6%) and intra-operative bleeding events (rigid 6%, flexible 8%) in adults. No clear superiority in efficacy could be depicted between flexible and rigid ETV for hydrocephalus treatment. Safety profiles varied by age but were acceptable for both techniques. Well-designed comparative studies are needed to assess the optimal endoscopic treatment option for hydrocephalus.

**Supplementary Information:**

The online version contains supplementary material available at 10.1007/s10143-021-01590-6.

## Introduction

Endoscopic third ventriculostomy (ETV) is a well-established surgical procedure for the treatment of hydrocephalus. It consists in the opening of the floor of the third ventricle using different types of tools driven through the operative channel of an endoscope [18]. The first ever-reported ETV was conducted by William J Mixter in 1923; he successfully treated a case of non-communicating hydrocephalus using an uretheroscope [18]. Ten years later, Tracy Putnam developed the “ventriculoscope,” the first endoscope specifically designed to operate in cerebral ventricles. It included one optical glass rod and three grooves, one for the light source and two for the diathermy electrodes [18]. The design and the optic systems, as well as the available operative tools, were then progressively refined. In the 1970s, the British physicist Harold H Hopkins with his system of solid and cemented glass rod lenses surrounded by fiberoptic bundles, paved the way for both the modern rigid and flexible endoscopy [18]. In 1973, Takanori Fukushima was the first neurosurgeon to use a flexible endoscope to perform ventriculostomies with his refined “ventriculofiberscope” [18].

Rigid and flexible endoscopes are both currently used to perform third ventriculostomy, and each type has distinct advantages and drawbacks. Rigid endoscopes are more commonly used compared with their flexible counterparts because they generally produce higher quality images and allow for easier passing of instruments [4]. Their use, however, can be restricted by the size of ventricles and made difficult by the rigid linear nature of the rod lenses [4, 5, 16, 38]. Flexible endoscopes, on the other hand, have an added degree of mobility to help overcome the nonlinear ventricular anatomy. They have been used more frequently in children given their narrower diameter, but they generally present images of lower quality and a limited set of operative tools [4, 5, 22]. Interestingly, the published literature usually focuses on the nuances and outcomes of either rigid or flexible endoscopy alone; only one paper compared the two techniques in a comparative study design to assess the optimal choice of treatment [57]. To our knowledge, no meta-analysis has been conducted to compare efficacy and safety of rigid endoscopy versus flexible endoscopy in ETV.

As the two approaches present both risks and benefits, we decided to pool the available evidence and conduct a meta-analysis to compare efficacy and safety of flexible and rigid neuro-endoscopy in the performance of ETV in pediatric and adult populations.

## Materials and methods

### Search strategy and study selection

A comprehensive electronic search was conducted on PubMED, EMBASE, and Cochrane until November 10, 2019. The search was filtered for English language articles. Comprehensive search results were obtained using relevant MeSH terms, Emtree terms, and text words (Appendix 1). The duplicates were removed and data were exported into Covidence software for screening [17]. All the articles underwent two levels of screening (title/abstract and full-text) by six reviewers (BM, AP, AB, FS, SD, AA). Discrepancies were resolved by discussion or consulting senior authors (AB, RM, FS). Reasons for rejection were listed in accordance with the PRISMA checklist [26].

### Inclusion and exclusion criteria

Articles were included in our study if: they had participants suffering from hydrocephalus who underwent flexible endoscopic third ventriculostomy or rigid endoscopic third ventriculostomy; the study reported failure or reoperation rate in the procedure; the study was an observational study, randomized control trial, or case series of five or more patients diagnosed with hydrocephalus. Articles were excluded from our study if they were not in the English language or if they did not report on patients’ outcome and follow-up.

### Data extraction

Studies included after full text screening had their data extracted by five authors (BM, AP, FS, SC, SD). Data were extracted for study characteristics (author, publication year, country of origin, study design and timing, and sample size), patients’ characteristics (average age, age category -pediatrics, adults-, type and etiology of hydrocephalus), and intervention characteristics (type of intervention and type of endoscope used). Efficacy or ETV failure was the primary outcome and was defined as patients requiring reoperations after ETV surgery which could either be a second ETV or shunt placement. Safety was assessed as a secondary outcome, evaluating incidence of complications including infection, intraventricular hemorrhage, neurological deficit, motor aphasia, ependymitis, sepsis, and CSF leak, among others, incidence of intra-operative bleeding (witnessed, controlled and reported by the operating surgeon), and incidence of death due to surgery. All the variables and outcomes were recorded for adults, pediatrics, and mixed (both pediatrics and adults) population. Number of events for failure and safety outcomes were recorded for each intervention.

### Data analysis

Incidence measures were analyzed for categorical outcomes by using number of events and total sample size of outcome measures. Pooled effect estimates of incidence measures were analyzed by the random-effects model using the DerSimonian–Laird method [26]. Comprehensive meta-analysis software (CMA) version 3 was used to perform the statistical analyses. Unless otherwise specified, a two-sided *p* value of < 0.05 was considered statistically significant.

### Heterogeneity assessment and analysis

The presence of heterogeneity was assessed using Cochrane Q statistic with a significance level of *p* < 0.10 [27]. Degree of heterogeneity among studies was determined using the I^2^ value [27]. Degree of heterogeneity was reported to be low, medium, and high with I^2^ values of 25, 50, and 75%, respectively [28]. All analyses were stratified by age categories (pediatric, adult, mixed). The *p* value comparing the subgroups was not derived as these would be highly confounded due to the nature of the included studies (non-comparative). An additional sensitivity analysis was done by removing low quality studies (< median score of 4) from all the analyses to assess the robustness of the findings.

### Risk of bias assessment

Publication bias was assessed by Begg’s [9] test and the funnel plot was analyzed for visual determination of asymmetry if the assessed outcomes had at least 10 studies [26]. If presence of publication bias was confirmed, the trim and fill method was used to estimate the possible number of missing studies, which were then imputed to recalculate the new pooled effect estimate. As all the studies included in the analysis were case series, the quality of the studies was assessed by a questionnaire by Chan and Bhanushali [14]. The questionnaire assessed all studies based on whether their objective, protocol, inclusion and exclusion criteria, time interval, and patient enrollment were well defined and if the studies had a prospective collection of outcome data and a high follow-up. Each category had one point associated to it with the highest possible score of 8. Studies with higher scores on the questionnaire were assessed to be of better quality.

## Results

### Search results and characteristics

**Fig. 1:**
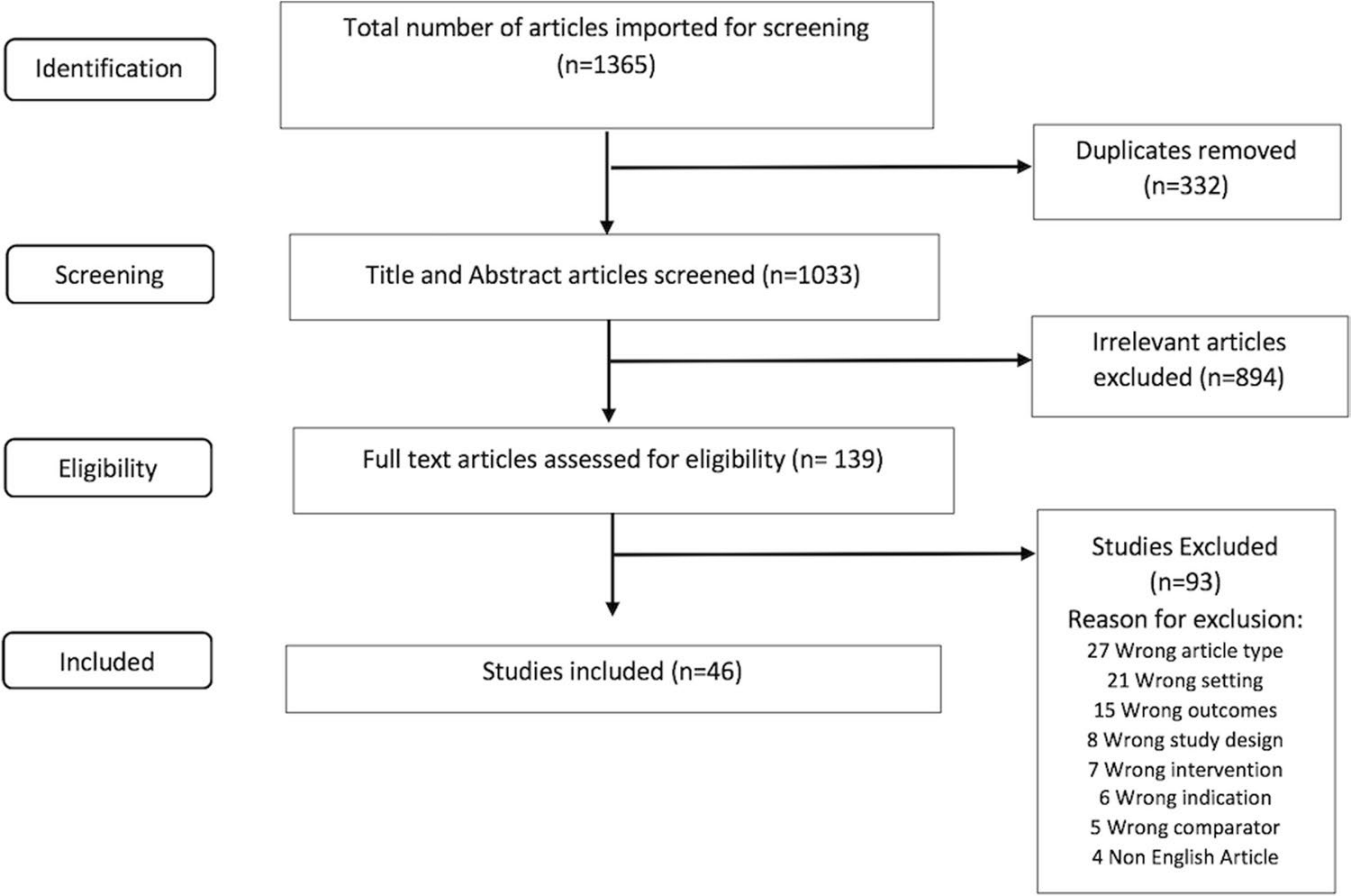
Study selection process of the identified articles

The electronic search yielded a total of 1365 studies [PubMed (743), EMBASE (602) and Cochrane (20)]. Of all imported studies, 1033 studies were screened and 46 case series [1–3, 6–8, 10–13, 15, 18–21, 23–25, 29, 30, 33–35, 37, 39–60] were used for the final meta-analysis (Fig. 1). The study timing for 39 studies was retrospective, while 7 studies were prospective. Patients in all age groups, from neonatal to geriatric population, were captured in the studies. The age range of the patients was 5 days–89 years and both naïve as well as previously shunted patients were included in the analysis. Out of the 46 case series with 3739 patients, 12 studies included adult population [7, 11, 13, 24, 25, 34, 35, 39, 40, 49, 50], 14 studies included pediatric population [1, 4, 7, 8, 12, 29, 33, 45, 46, 51, 58, 60], and 20 studies included patients from both groups [2, 10, 15, 19, 21, 23, 29, 30, 37, 41–44, 47, 52, 53, 55, 56, 59]. Regarding flexible ETV, 10 studies [23, 34, 35, 41, 42, 48, 52, 53, 57, 58] reported outcomes with a total of 821 patients, of whom 38 were adults, 126 were pediatric, and 657 were a mixture of adult and pediatric populations. For rigid ETV, 37 studies [1–4, 6–8, 10–13, 15, 18–21, 24, 25, 29, 30, 33, 37, 39, 40, 43–47, 49–51, 54–57, 59, 60] reported outcomes for a total of 2918 patients, of whom 1018 were adults, 747 were pediatric, and 1153 patients were a mixture of adult and pediatric populations. The types of hydrocephalus included were communicating hydrocephalus, non-communicating hydrocephalus, and normal pressure hydrocephalus (Table 1).

**Table 1:** Characteristics of studies included in the systematic review and meta-analysis

Author, Year	Endoscope (Rigid/Flexible)	Study Design, Timing	Hydrocephalus type	Etiology (no. of patients)	Patient Population	Age: Mean (range) unless otherwise specified	Male, n (%)	Study Quality*
Abbassy et al., 2018 [1]	Rigid	Case series, retrospective	Non-communicating hydrocephalus	Endodermal sinus tumor (1), Germinoma (2), Grade II astrocytoma (1), Mixed germ-cell tumor (1), Pineoblastoma (3), Pilocytic astrocytoma (1), Grade II tectal glioma (1), Pilocytic astrocytoma (1)	Pediatric	11 years (1 year–17 years)	10 (90.9%)	3
Aranha et al., 2018 [3]	Rigid	Case series, prospective	Communicating hydrocephalus	Tuberculous meningitis (TBM) Hydrocephalus	Pediatric	NR	15 (57.7%)	4
Chiba et al., 2018 [15]	Rigid	Case series, retrospective	Non-communicating hydrocephalus	Aqueductal stenosis (3), Cerebellar tumor (2), Intraventricular hemorrhage (3), Pineal tumor (13), Fourth ventricle tumor (2), Brainstem tumor (1), Tectal tumor (3), Brain metastasis (1), Isolated fourth ventricle (1), Bilateral thalamic glioma (1)	Adult and Pediatric	31.4 years (0.3 years–74 years)	17 (56.7%)	5
Torres-Corzo et al., 2018 [52]	Flexible	Case series, retrospective	Non-communicating hydrocephalus	NR	Mixed	20.45 years	25 (52%)	4
Uche et al., 2018 [54]	Rigid	Case series, prospective	Non-communicating hydrocephalus	Aqueductal stenosis (37), Dandy-Walker malformation (7), Arnold-Chiari malformation (8), Intraventricular cyst loculations (3)	Pediatric	2.3 years (3 months–4.5 years)	37 (67%)	5
Wu et al., 2018 [59]	Rigid	Case series, retrospective	Non-communicating hydrocephalus	Chiari type I malformation (10)	Adult and Pediatric	28.14 years (0.75 years–55 years)	4 (40%)	5
Sufianov et al., 2018 [51]	Rigid	Case series, prospective	Non-communicating hydrocephalus	Aqueductal stenosis (21), Post hemorrhagic hydrocephalus (25), Post-infectious hydrocephalus (11), Dandy-Walker malformation (4), Myelomeningocele (3)	Pediatric	NR	34 (53.1%)	4
Aref et al., 2017 [4]	Rigid	Case series, retrospective	Nr	Normal pressure hydrocephalus (40), Obstructive tumor (46), Aqueductal stenosis (18), Hemorrhage (3), Unknown etiology (19), Trauma (3), Tuberculosis (1), Cyst (7)	Adult	51.7 years	82 (62%)	4
Oertel et al., 2017 [43]	Rigid	Case series, retrospective	Non-communicating hydrocephalus	Benign aqueductal stenosis (40), Tumor (35), Infection/hemorrhage (15), Intracranial cysts (13), Other (10)	Adult and Pediatric	NR	58 (51.3%)	2
Wang et al., 2017 [57]	Rigid	Case series, retrospective	Nr	Post-hemorrhagic hydrocephalus (25), Aqueductal stenosis (21), Myelomeningocele (23), Dandy-Walker complex (6), Congenital idiopathic hydrocephalus (4), Postinfectious hydrocephalus (6), Other etiology (6)	Pediatric	Median = 3.5 months IQR = 1.2 months–5.7 months	45 (52.9%)	5
	Flexible	Case series, retrospective	Nr	Post-hemorrhagic hydrocephalus (44), Aqueductal stenosis(12), Myelomeningocele(7), Dandy-Walker malformation (6), Congenital idiopathic hydrocephalus (6), Postinfectious hydrocephalus (5), Other etiology(5)	Pediatric	Median = 3.2 months IQR = 0.7 months—6.8 months	50 (54.9%)	5
Rodriguez et al., 2017 [58]	Flexible	Case series, retrospective	Nr	Tumor (20), infectious or due to parasites (75), Post-hemorrhagic (16), Congenital (39)	Mixed	(4 days–76 years)	76 (51%)	5
Zhao et al., 2016 [60]	Rigid	Case series, retrospective	Non-communicating hydrocephalus (24), communicating hydrocephalus (13)	Posterior fossa tumor or pineal tumor (9), Idiopathic aqueduct stenosis (15), Post-meningitis (8), Post-hemorrhagic (3)	Pediatric	(8 months–36 months) Median = 21.6 months	NR	4
Grand et al., 2016 [24]	Rigid	Case series, retrospective	Nr	Aqueduct stenosis (56), Intraventricular hemorrhage (20), Remote head trauma (57), Post-craniotomy for posterior fossa tumor (14), Subarachnoid hemorrhage (23), Tumor or cyst (42), Shunt obstruction (23), Other cause (8)	Adult	51 years (17 years–88 years)	128 (52.7%)	4
Kawsar et al., 2015 [30]	Rigid	Case series, retrospective	Nr	Aqueductal stenosis (210), Posterior fossa tumors (74), Cysts (56), Previous ventriculitis (49), Shunt failure (8), Posterior fossa hemorrhage (6), Hydrocephalus due to Chiari malformation (6), Congenital fourth ventricular outflow obstruction (2) Hydrocephalus w/ empty sella syndrome (1)	Mixed	NR	224 (54.37%)	4
Niknejad et al., 2015[39]	Rigid	Case series, retrospective	Tri-ventricular hydrocephalus (14), communicating hydrocephalus (1), quadri-ventricular hydrocephalus (1)	Tumor (8), Giant basilary tip aneurysm (1), Normal pressure hydrocephalus (1), idiopathic aqueductal stenosis (3), post-hemorrhagic (1), Tuberculous meningitis (1), Wegener granuloma (1)	Adult	72.8 years (66 years–83 years)	11 (68.75%)	5
Obaid et al., 2015 [40]	Rigid	Case series, retrospective	Non-communicating hydrocephalus	Intraventricular hemorrhage	Adult	58 years (42 years–79 years)	9 (52.94%)	5
Vulcu et al., 2015 [56]	Rigid	Case series, retrospective	Nr	Benign aqueductal stenosis (40), Tumor (35), Infection/hemorrhage (15), Intracranial cysts (13), Other (10)	Mixed	35 years (8 days–77 years)	58 (51.3%)	4
Bisht et al., 2014 [10]	Rigid	Case series, retrospective	Non-communicating hydrocephalus	Congenital aqueductal stenosis	Adult and Pediatric	7.45 years (1 month–52 years)	79 (77.45%)	6
Salvador et al., 2014 [47]	Rigid	Case series, retrospective	Non-communicating hydrocephalus	Congenital malformations (74), Tumor (52), Cysts (15), Infection (12), Hemorrhage (11)	Adult and Pediatric	22.1 years	98 (60%)	6
Stachura et al., 2014 [50]	Rigid	Case series, retrospective	Non-communicating hydrocephalus	Primary aqueductal stenosis (24), Brain tumor (61), Basilar tip aneurysm (2), Undetermined (9)	Adult	47 years (18 years–82 years)	55 (57.3%)	2
Ali et al., 2013 [2]	Rigid	Case series, retrospective	Non-communicating hydrocephalus	Posterior fossa tumor (83), Aqueductal stenosis (37), Non tectal tumor (8), CP angle tumor (7), Tectal tumor (7), Posterior fossa abscess (3), Posterior fossa hematoma (2)	Mixed	15 years (6 months–60 years)	72 (46.45%)	5
Brusius & Cavalheiro, 2013 [12]	Rigid	Case series, prospective	Non-communicating hydrocephalus	Blake pouch cyst (8)	Pediatric	13.25 months (1 month–48 months)	5 (62.5%)	7
Melot et al., 2013 [37]	Rigid	Case series, retrospective	Non-communicating hydrocephalus	Malformation (43), Mass lesion (35), Post-infectious (4)	Adult and Pediatric	NR	NR	4
Romeo et al., 2013 [46]	Rigid	Case series, retrospective	Non-communicating hydrocephalus	Tectal plate gliomas	Pediatric	11.6 years (4 years–18 years)	15 (68%)	3
Vogel et al., 2013 [55]	Rigid	Case series, retrospective	Non-communicating hydrocephalus	Aqueductal stenosis (35), Non-tectal tumor (23), Tectal tumor (16), Myelomeningocele (9), Intracranial cyst (6), Infection (3), Chiari malformation Type I (3)	Mixed	19.7 years (5 months–77 years)	46 (48%)	4
Bouramas et al., 2012 [11]	Rigid	Case series, retrospective	Non-communicating hydrocephalus	Aquetuctal stenosis (30), Post infection (3), Cyst (5), Tumor (15), Hemorrhage (4)	Adult	(43 years–89 years)	26 (48.14%)	5
Warf et al., 2012 [58]	Flexible	Case series, retrospective	Non-communicating hydrocephalus	Congenital aqueductal stenosis	Pediatric	NR	19 (54.28%)	4
Durnford et al., 2011 [19]	Rigid	Case series, retrospective	Non-communicating hydrocephalus	Aqueductal stenosis (40), Non-tectal tumor (39), Intraventricular Hemorrhage (23), Tectal tumor (15), Myelomeningocele (5), Post-infectious (7), Other (37)	Mixed	(0 years–19 years)	95 (57.2%)	4
Egger et al., 2010 [20]	Rigid	Case series, retrospective	Non-communicating hydrocephalus	Cyst (4), Tumor (4), Chiari II Malformation and myelomeningocele (3), Aqueductal stenosis (3)	Pediatric	Median = 3 years 4 months	6 (42.8%)	5
Ogiwara et al., 2010 [45]	Rigid	Case series, retrospective	Non-communicating hydrocephalus	Congenital aqueduct stenosis (11), Post-hemorrhagic obstruction (6), Myelomeningocele (2), Post-meningitis (2), Chiari I malformation (1), Dandy walker variant (1)	Pediatric	87.7 days (5 days–158 days)	14 (60.8%)	4
Torres-Corzo et al., 2010 [53]	Flexible	Case series, retrospective	Non-communicating hydrocephalus	Neurocysticercosis (86)	Mixed	(9 years–79 years)	42 (56%)	4
Oertel et al., 2009 [44]	Rigid	Case series, prospective	Non-communicating hydrocephalus	Cerebellar hemorrhage (17), Thalamic hemorrhage (6), Intraventricular hemorrhage (5), basal ganglia hemorrhage (3), subarachnoid hemorrhage (2), pontine hemorrhage (1)	Mixed	60.8 years (3 months–83 years)	15 (44%)	6
Ersahin & Arslan, 2008 [21]	Rigid	Case series, retrospective	Non-communicating hydrocephalus	Obstruction of fourth ventricular outlets, Dandy-Walker Malformation, Chiari Malformation type I, Chiari malformation type II, Aqueductal stenosis	Mixed	14.8 years (2 months–77 years)	85 (54.8%)	5
Hailong et al., 2008 [25]	Rigid	Case series, retrospective	Idiopathic normal pressure (17), secondary communicating hydrocephalus (15)	Idiopathic normal-pressure hydrocephalus (17), Tubercular meningitis (1), Trauma (9), Hypertensive intracranial hemorrhage (4), Subarachnoid hemorrhage (1)	Adult	61.4 years	24 (75%)	3
Lipina et al., 2008 [33]	Rigid	Case series, retrospective	Non-communicating hydrocephalus	Acqueductal stenosis (5), Peri and intraventricular hemorrhage (8), Hemorrhage and Infection (1)	Pediatric	105 days	8 (57.1%)	5
Idowu et al., 2008 [29]	Rigid	Case series, prospective	Non-communicating hydrocephalus	Aqueductal stenosis (11), Dandy-Walker malformation (9), Myelomeningocele (4), Pineal region tumor(1)	Mixed	(4 weeks to 48 years) median = 6 months	14 (56%)	4
Baldauf et al., 2007 [7]	Rigid	Case series, retrospective	Non-communicating hydrocephalus	Idiopathic aqueductal stenosis (8), Other congenital anomalies (4), Post-hemorrhagic (3), Tumor-related (3), Shunt infection (2), Shunt failure (1)	Pediatric	6.7 months	15 (71.4%)	3
Baldauf et al., 2006 [6]	Rigid	Case series, retrospective	Non-communicating hydrocephalus	cerebellar infarction	Adult	62 years (25 years–85 years)	5 (55%)	4
O'Brien et al., 2006 [41]	Flexible	Case series, retrospective	Non-communicating hydrocephalus	NR	Mixed	37 years (5 years–77 years) Median = 33	21 (50%)	3
Baykan et al., 2005 [8]	Rigid	Case series, retrospective	Non-communicating hydrocephalus	NR	Pediatric	(2 months—10 years)	120 (57.1%)	5
O'Brien et al., 2005 [42]	Flexible	Case series, retrospective	Non-communicating hydrocephalus	Spina bifida, aqueductal stenosis, arachnoidi cysts, primary infective and haemorrhagic origin	Mixed	27.78 (37 weeks–77 years)	NR	4
	Flexible	Case series, retrospective	Non-communicating hydrocephalus	Spina bifida, aqueductal stenosis, arachnoidi cysts, primary infective and haemorrhagic origin (IVH)	Mixed	20.43 (9 months–69 years)	NR	4
Santamarta et al., 2005 [49]	Rigid	Case series, retrospective	Non-communicating hydrocephalus	Primary aqueductal stenosis (27), Tumoural (30), Non tumoural (haemorrhage, cysts) (9)	Adult	Median = 53 IQR = 27–67	33 (50%)	5
Longatti et al., 2004 [34]	Flexible	Case series, retrospective	Nr	Primitive aqueductal stenosis (3) secondary CSF pathway obstruction (11) frontal cystic glioblastoma (1) normal pressure hydrocephalus (6) previously shunted (3)	Adult	(35 years–82 years) Median = 59.5	14 (58.3%)	5
Longatti et al., 2004 [35]	Flexible	Case series, retrospective	Normal pressure hydrocephalus	NR	Adult	(66 years–78 years)	8 (57.1%)	5
Buxton et al., 2001 [13]	Rigid	Case series, retrospective until august 1994, post aug 1994 prospective	Non communicating hydrocephalus (44), communicating hydrocephalus (9)	Third ventricular tumor (22) Aqueduct stenosis (18), Third ventricular arachnoid cyst (4) Infection (4), SAH/Post haemorrhagic (2) Myelomeningocoele (4), Other (4) Hydrocephalus cause (3)	Adult	37.5 years (17 years–77 years)	38 (60.3%)	7
Gangemi et al., 1999 [23]	Flexible	Case series, retrospective	Triventricular hydrocephalus (110), tetraventricular hydrocephalus (15)	Primary aqueductal stenosis (77) mesencephalic tumors (16), pineal region tumors (9) posterior fossa tumors (8) blockage in the posterior fossa (11) subarachnoid hemorrhage (3), infection due to Candida (1)	Mixed	31 years (7 days–81 years)	72 (57.6%)	6

^*^ Study quality (Median 4, IQR 4–5) was assessed based on the quality assessment questionnaire for case series based on Chan and Bhandari

### Efficacy (ETV failure) analysis

**Fig. 2:**
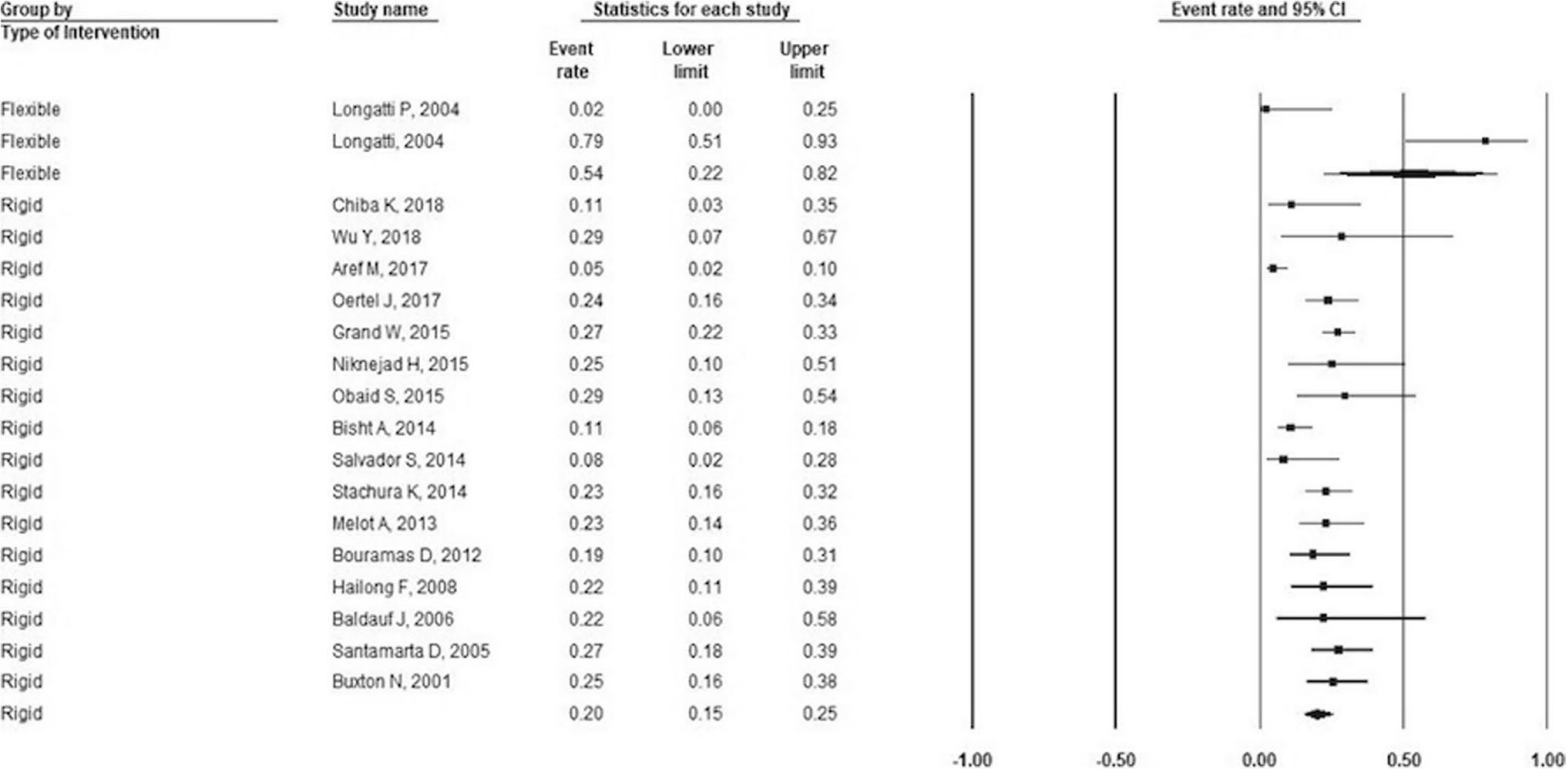
Forest plot for incidence of failure in adults stratified by endoscopy type. For flexible ETV: incidence of failure = 54%; number of studies = 2; P-heterogeneity = 0.001; I^2^ = 90.9%; for rigid ETV: incidence of failure: 20% number of studies = 16; P-heterogeneity = 0.002; I^2^ = 57.4%. Error bars represent the 95% CI. ETV: endoscopic third-ventriculostomy

**Fig. 3:**
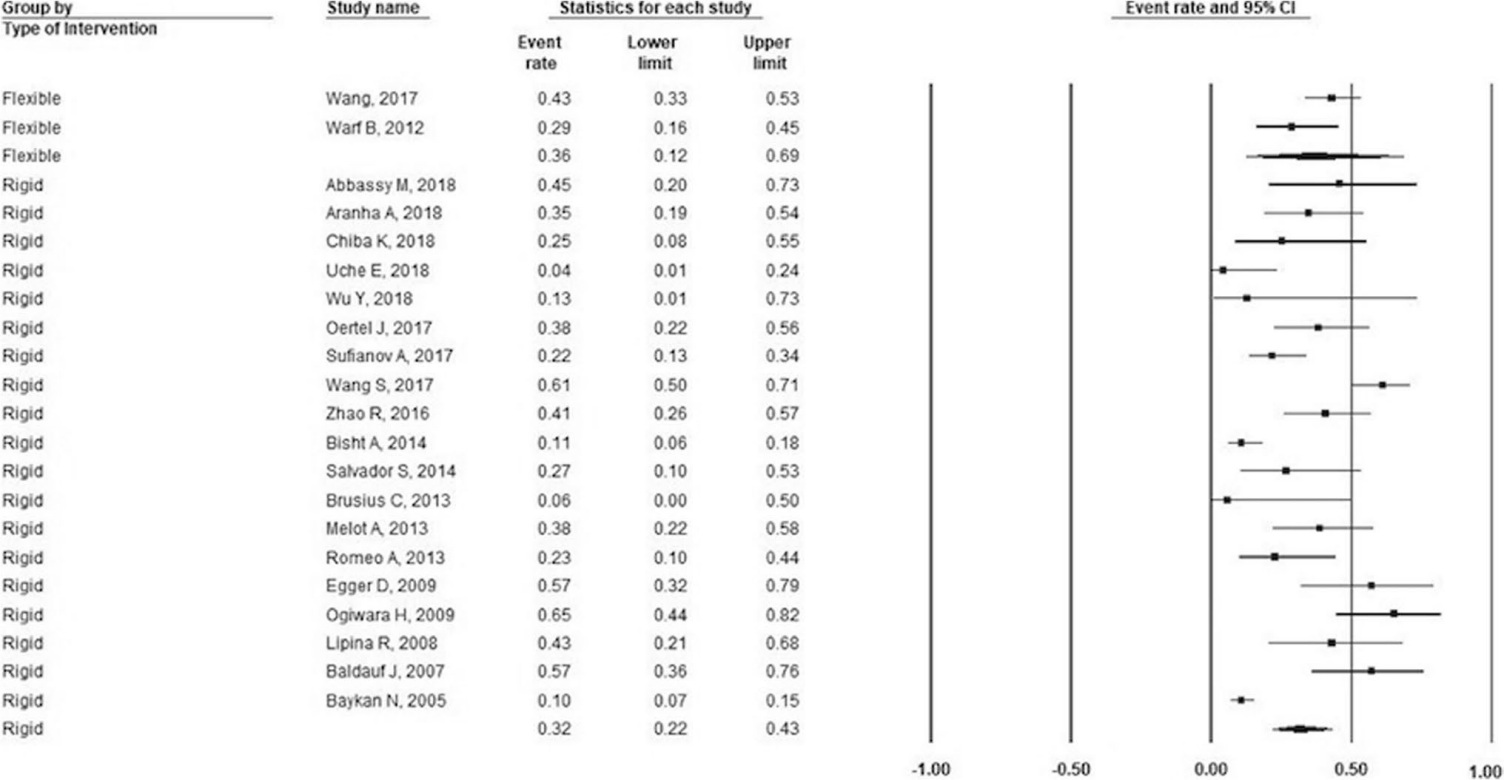
Forest plot for incidence of failure in pediatric population stratified by endoscopy type. For flexible ETV: incidence of failure = 36%; number of studies = 2; P-heterogeneity = 0.14; I^2^ = 53.2%; for rigid ETV: incidence of failure = 32%; number of studies = 19; P-heterogeneity = 0.00; I^2^ = 85.2%. Error bars represent the 95% CI. ETV: endoscopic third-ventriculostomy

**Fig. 4:**
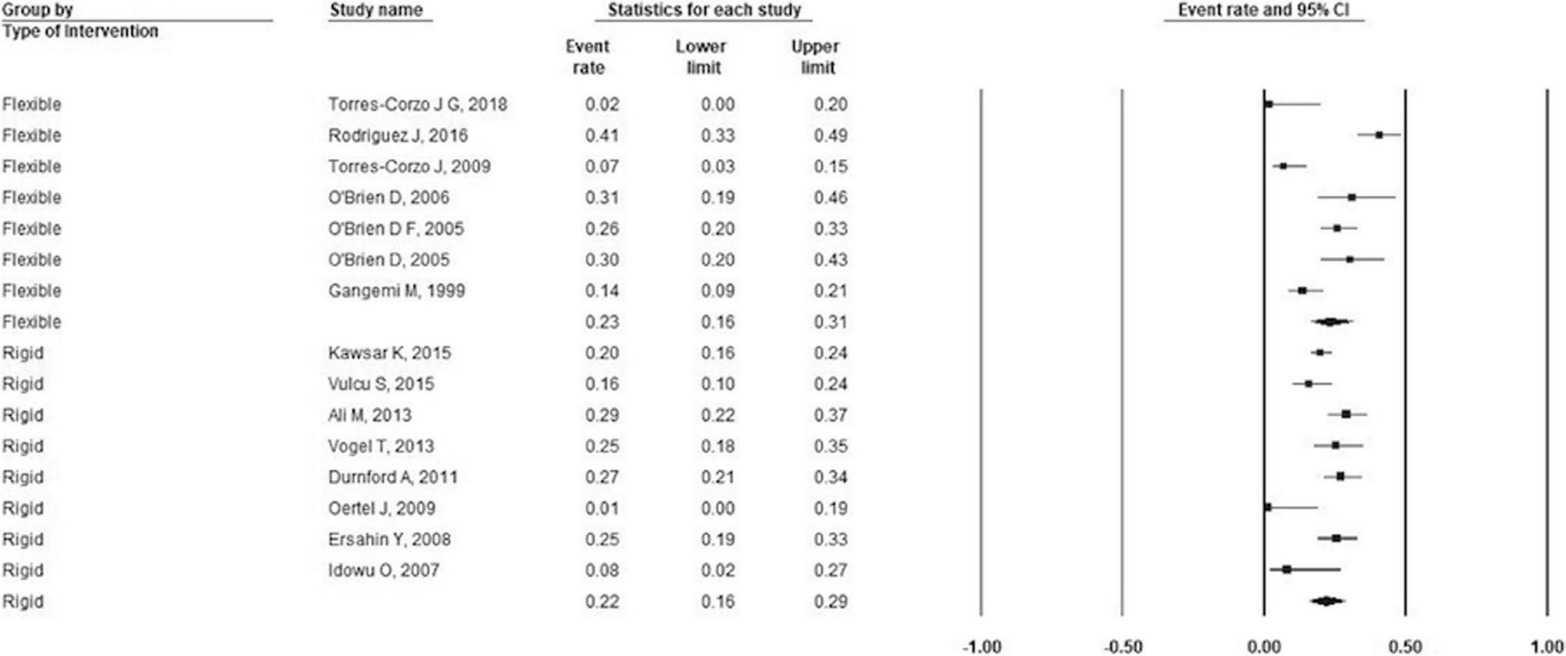
Forest plot for incidence of failure in mixed population stratified by endoscopy type. For flexible ETV: incidence of failure = 23%; number of studies = 7; P-heterogeneity = 0.00; I^2^ = 86%; for rigid ETV: incidence of failure = 22%; number of studies = 8; P-heterogeneity = 0.01; I^2^ = 61%. Error bars represent the 95% CI. ETV: endoscopic third-ventriculostomy

Flexible ETV showed a higher incidence of failure compared with rigid ETV in adults (54% vs 20%) (Fig. 2), while a smaller difference was found in pediatric patients (36% flexible vs 32% rigid) (Fig. 3) and mixed age patients (23% flexible vs 22% rigid) (Fig. 4) (Table 2).

**Table 2:** Pooled effect estimates for efficacy (failure)

		Flexible ETV			Rigid ETV		
Outcome	Population type;	Pooled incidence (95% C.I.)	I^2^ value	# of studies	Pooled incidence (95% C.I.)	I^2^ value	# of studies
Failure	Pediatric	36% (12%, 66%)	53.2%	2	32% (22%, 43%)	85.2%	19
	Adult	54% (22%, 82%)	90.9%	2	20% (15%, 25%)	57.4%	16
	Mixed	23% (16%, 31%)	86%	7	22% (16%, 27%)	61.6%	8

C.I.: Confidence interval

### Safety analysis (complications, bleeding, death)

Even though pooled results could not be compared with a statistical *p* value, it was worth exploring the trends resulting from our analysis. Flexible endoscopy presented an overall lower incidence of complications in pediatric (2 vs 18%) and mixed populations (8 vs 11%) but not in adults (13 vs 9%) when compared with the rigid approach (Table 3, Appendix 2). Flexible endoscopy presented an overall trend towards lower incidence of intra-operative bleeding in the mixed age category (4 vs 6%) but not in the adult category (8 vs 6%) when compared with the rigid approach**.** No studies conducted in pediatrics presented data on intra-operative bleeding (Table 3, Appendix 3). Flexible endoscopy reported lower incidence of death related to surgery in each age group (pediatric 1 vs 3%, adult 4 vs 6%, mixed 1.2 vs 1.7%) when compared with the rigid approach (Table 3, Appendix 4).

**Table 3:** Pooled effect estimates for safety outcomes of complications, bleeding, and death

		Flexible ETV			Rigid ETV		
Outcome	Population;	Pooled incidence (95% C.I.)	I^2^ value	# of studies	Pooled incidence (95% C.I.)	I^2^ value	# of studies
Complications	Pediatric	2% (0.1–34%)	N.A	1	18% (7–41%)	90.8%	7
	Adult	13% (3–40%)	N.A	1	9% (5–13%)	55.9%	8
	Mixed	8% (5–11%)	54%	5	11% (8–16%)	27.9%	4
Bleeding	Adult	8% (2–27%)	N.A	1	6% (4–10%)	0%	4
	Mixed	4% (2–9%)	19.1%	5	6% (2–13%)	89.7%	4
Death	Pediatric	1% (0.1–3%)	0%	2	3% (1–5%)	0%	12
	Adult	4% (0.4–32%)	0%	2	6% (2–13%)	80.9%	10
	Mixed	1.2% (0.6–2.7%)	0%	6	1.7% (1.1–2.8%)	0%	7

*CI* confidence interval, *NA* not applicable

### Quality score and bias assessment

The quality score for all studies ranged from 2 to 7 with a median score of 4 (IQR 4–5) (Appendix 5) on the Chan and Bhanushali questionnaire. Only 7 studies had a quality score < median [1,7,25,41,43,46,50,]. All studies had a well-defined study objective and clinically relevant outcomes. The majority of them had well-defined protocols and high follow-up rates. A few studies did not report explicit inclusion/exclusion criteria, time interval, and consecutive patient enrollment. Only seven studies had prospective data collection. Only the rigid endoscopy group with regard of the incidence of failure had more than 10 studies in their analysis for each of the adult and pediatric populations.

**Fig. 5:**
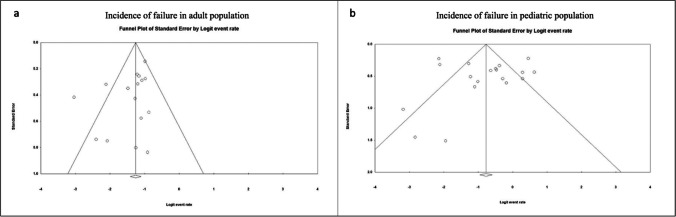
Funnel plots for incidence of failure in adult and pediatric populations undergoing rigid ETV. No evident signs of asymmetry are unveiled in adult (**a**) or pediatric (**b**) population. The Begg’s test confirmed these findings (adult *p* value 0.22, pediatric *p* value 0.55). ETV: endoscopic third-ventriculostomy

The funnel plot for the incidence of failure using the rigid endoscopy did not show obvious signs of asymmetry in adult population (Fig. 5a) or pediatric population (Fig. 5b), which suggested the absence of publication bias. The Begg’s test for each was not statistically significant, further confirming these findings (*p* value: 0.22 in adults; *p* value: 0.55 in pediatrics).

### Sensitivity analysis

All of the above analyses did not materially change when we excluded studies with a quality score below the median level (< 4) (Appendices 6 and 7).

## Discussion

The results of this meta-analysis suggested the presence of better efficacy of rigid endoscopy for ETV performance in adults. Safety profiles were mixed, while flexible endoscopy showed fewer complications in pediatrics and lower death events in pediatrics and adults, rigid endoscopy showed fewer complications and bleeding events in adults.

Regarding the efficacy profile, the results for the adult group were limited by the availability of only two studies on flexible endoscopy [34, 35]. It is particularly important to notice that one of these two studies focused on patients suffering from normal pressure hydrocephalus, which is known to have overall better outcomes when treated with a shunt [35], given the non-obstructive nature of the disease [36]. Therefore, the efficacy results were more suggestive of the fact that ETV was able to provide actual benefit to patients with hydrocephalus depending on its etiology, rather than providing evidence of an overall superiority of flexible or rigid approach over the other. The available literature has in fact already shown that both etiology and age are crucial factors to consider in the decision of treating hydrocephalus through a shunt or ETV, particularly in the pediatric population [31, 32].

In terms of safety, both flexible and rigid endoscopic approaches turned out to be procedures with acceptable peri-operative complication rates and very low occurrence of intra-operative bleeding and death. With regard to peri-operative complications, we could appreciate a trend towards a lower rate in the use of flexible approach, particularly in the pediatric population, but whether these comparisons would reach statistical significance is yet to be confirmed in future comparative studies. Flexible instruments are smaller and tend to be more delicate, which could at least in part explain our findings. With regard to intra-operative bleeding, the results need to be interpreted cautiously. The risk of bleeding depends also on the type of procedure performed during the endoscopy: a patient who undergoes ETV alone has a reduced risk of experience bleeding compared to a patient who undergoes ETV along with the biopsy or partial resection of a tumor or again the cauterization of the choroid plexus, regardless the type of approach. Interestingly, no pediatrics study reported occurrence of intra-operative bleeding, even in the presence of choroid plexus cauterization. Moreover, the ability of the flexible endoscope to reach areas out of range for the rigid one, for example, the posterior half of the third ventricle, allows the surgeon to perform deeper maneuvers, hence exposing them to the related inherent risks. Regardless the approach and age group, intraoperative mortality was found to be a very rare event, confirming both flexible and rigid endoscopy as safe techniques.

The I^2^ value for most groups was reported to be high. The degree of heterogeneity could be explained by to the presence of other co-variates such as the type of hydrocephalus (communicating, non-communicating, and normal pressure hydrocephalus) and its etiology; however, we could not assess their effect in the determination of the results due to lack of data. Notably, study quality was not found to be a source of heterogeneity as the results were not altered after excluding the low-quality studies.

In the interpretation of the results of this study, a number of limitations needs to be taken into account. First, the presence of reporting imbalance in the two techniques; out of all the studies that were included in the final analysis, only 10 studies reported data on flexible ETV, while 36 studies reported data on rigid ETV. The study design consisted of case series and no other comparative studies. Due to the lack of randomized control trials or comparative (analytical) observational studies in the meta-analysis, results need to be interpreted with caution due to possible confounding bias and other biases typically present in case series. Hence, the *p* values comparing the pooled point estimates between the 2 techniques were not derived. A major challenge faced while conducting the study was that only one study (Wang et.al) [57] had data for both intervention arms directly compared in a propensity-score matched cohort study, which were included as separate groups in this analysis. The study included only pediatrics and reported that rigid endoscopy had worse outcomes of failure as compared with flexible endoscopy, which was discordant with our findings. This begs the need for more well-designed studies in pediatrics and adults in order to accurately discern these differences. Notably, the type of hydrocephalus and its etiology could not be taken into account in the analysis due to lack of data, whereas in clinical practice, these two factors are part of the decision-making process in the choice of treatment strategy. Regardless, our aim was to evaluate efficacy and safety of two approaches that are both endoscopic in nature, therefore specific considerations about indications for alternative treatments as, for example, shunt diversion, were out the scope of this work.

Despite these limitations, our study had some strengths. To our knowledge, this was the first meta-analysis performed with the aim to evaluate efficacy and safety of flexible vs rigid ETV for the treatment of hydrocephalus. Another strength is the stratification of all safety and efficacy outcomes by age category, while shedding light on the available data in the entire neurosurgery literature and suggesting steps needed for better designed studies to address some uncertainties.

In conclusion, while our analysis could not depict a clear superiority in terms of efficacy with regard to flexible vs rigid endoscopy in the treatment of hydrocephalus, our results suggested that both approaches presented acceptable safety profiles, with some degree of variability between age categories. Moving forward, well-designed randomized controlled trials and comparative observational studies with larger sample sizes including patients of different ages, types, and etiology of hydrocephalus are needed in order to assess the optimal treatment options between rigid ETV and flexible ETV for hydrocephalus treatment.

## Supplementary Information

Below is the link to the electronic supplementary material.

Supplementary file1 (DOCX 721 KB)

## Data Availability

Not applicable.
