# Supplementary material for: Efficacy and safety of flexible versus rigid endoscopic third ventriculostomy in pediatric and adult populations: a systematic review and meta-analysis
Source: Neurosurg Rev. 2021 Jun 25;45(1):199–216. doi: 10.1007/s10143-021-01590-6 (PMC8827229; doi:10.1007/s10143-021-01590-6)
Supplement: Supplementary file 1 — Supplementary file1 (DOCX 721 KB) [file 10143_2021_1590_MOESM1_ESM.docx]

**Appendix 1. Search Terms**

**PubMed**

(("Hydrocephalus"[Mesh] OR Hydrocephalus [tw] OR noncommunicating hydrocephalus [tw] or non-communicating hydrocephalus[tw] OR obstructive hydrocephalus [tw] OR Hydrocephaly[tw] OR Cerebral Ventriculomegal*[tw] or Aqueductal Stenos*[tw]) AND (flexible neuroendoscop* [tw] OR flexible endoscop* [tw]) AND ("Ventriculostomy"[Mesh] or Ventriculostomy [tw] or Endoscopic Third Ventriculostomy [tw] or ETV* [tw] or Ventriculocisternostom*[tw] ))

OR

(("Hydrocephalus"[Mesh] OR Hydrocephalus [tw] OR noncommunicating hydrocephalus[tw] or non-communicating hydrocephalus [tw] OR obstructive hydrocephalus [tw] OR Hydrocephaly[tw] OR Cerebral Ventriculomegal*[tw] or Aqueductal Stenos*[tw]) AND (rigid neuroendoscop* [tw] OR rigid endoscop* [tw] or neuroendoscop* [tw] OR cerebral endoscop* [tw] or "Neuroendoscopy"[Mesh] or ventriculoscop*[tw] OR neurologic endoscop*[tw]) AND ("Ventriculostomy"[Mesh] or Endoscopic Third Ventriculostomy [tw] or ETV* [tw] OR Ventriculocisternostom*[tw] or ventriculostomy [tw]))

# of Articles: 743 on 11/10/2019

**Embase**

((exp hydrocephalus/ OR (Hydrocephalus OR noncommunicating hydrocephalus or non-communicating hydrocephalus OR obstructive hydrocephalus OR Hydrocephaly OR Cerebral Ventriculomegal* or Aqueductal Stenos*).tw. ) AND (third ventriculostomy/ OR (Endoscopic Third Ventriculostom* or ETV* OR Ventriculocisternostom* or third ventriculostom*).tw.) AND ( (flexible neuroendoscop* OR flexible endoscop*).tw))

OR

((exp hydrocephalus/ OR (Hydrocephalus OR noncommunicating hydrocephalus or non-communicating hydrocephalus OR obstructive hydrocephalus OR Hydrocephaly OR Cerebral Ventriculomegal* or Aqueductal Stenos*).tw. ) AND (third ventriculostomy/ OR (Endoscopic Third Ventriculostom* or ETV* OR Ventriculocisternostom or third ventriculostom*).tw.) AND ( (rigid neuroendoscop* OR rigid endoscop* or neuroendoscop* OR cerebral endoscop* or ventriculoscop* OR neurologic endoscop*).tw. or neurological endoscope/ OR neuroendoscopy/))

**# of Articles: 602 on 11/10/2019**

**Cochrane**

(Hydrocephalus[Mesh] OR (Hydrocephalus OR noncommunicating hydrocephalus or non-communicating hydrocephalus OR obstructive hydrocephalus OR Hydrocephaly OR Cerebral Ventriculomegal* or Aqueductal Stenos*):ti,ab,kw) AND (Ventriculostomy[Mesh] or (Endoscopic Third Ventriculostomy or ETV* or Ventriculocisternostom* or Ventriculostomy):ti,ab,kw) AND ((flexible neuroendoscop* OR flexible endoscop*):ti,ab,kw)

OR

(Hydrocephalus[Mesh] OR (Hydrocephalus OR noncommunicating hydrocephalus or non-communicating hydrocephalus OR obstructive hydrocephalus OR Hydrocephaly OR Cerebral Ventriculomegal* or Aqueductal Stenos*):ti,ab,kw) AND (Ventriculostomy[Mesh] or (Endoscopic Third Ventriculostomy or ETV* or Ventriculocisternostom* or Ventriculostomy):ti,ab,kw) AND (Neuroendoscopy [mesh](rigid neuroendoscop* OR rigid endoscop* or neuroendoscop*):ti,ab,kw)

# of Articles: 20 on 11/10/2019

**Total: 1365 on 11/10/2019**

**Appendix 2. Forest plot for incidence of complications**

**Adult population**
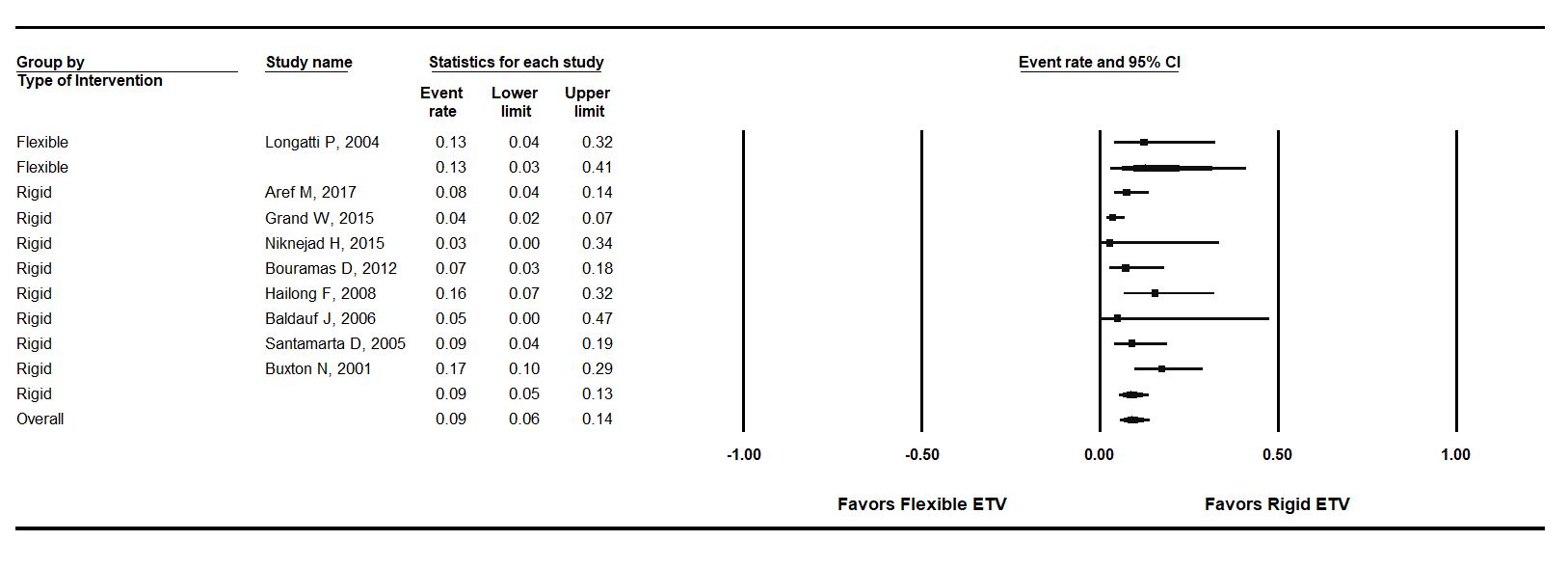
Forest plot for incidence of complication in adults stratified by endoscopy type. For flexible ETV: incidence of failure = 13%; number of studies = 1; P-heterogeneity = 1.00; I^2^ = N.A.; for rigid ETV: incidence of failure: 9% number of studies = 8; P-heterogeneity = 0.026; I^2^ = 55.90%. P-value comparing flexible to rigid = 0.62. Error bars represent the 95% CI. ETV: endoscopic third-ventriculostomy

**Pediatric population**


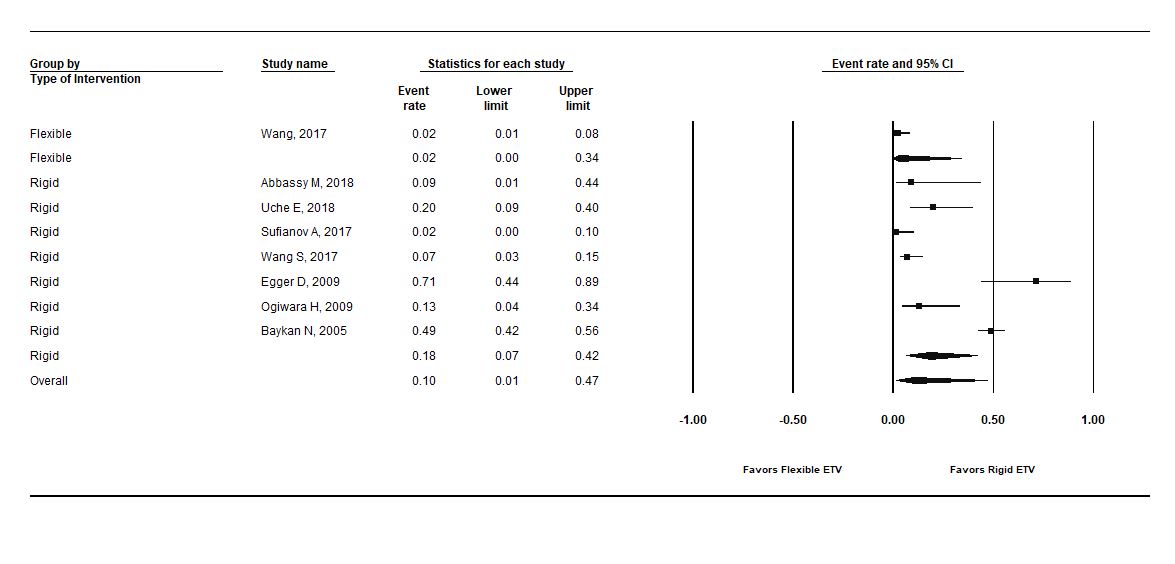


Forest plot for incidence of complication in pediatric population stratified by endoscopy type. For flexible ETV: incidence of failure = 2%; number of studies = 1; P-heterogeneity = 1.00; I^2^ = N.A.; for rigid ETV: incidence of failure: 18% number of studies = 7; P-heterogeneity = 0.0; I^2^ = 90.80%. P-value comparing flexible to rigid = 0.18. Error bars represent the 95% CI. ETV: endoscopic third-ventriculostomy

**Mixed population**


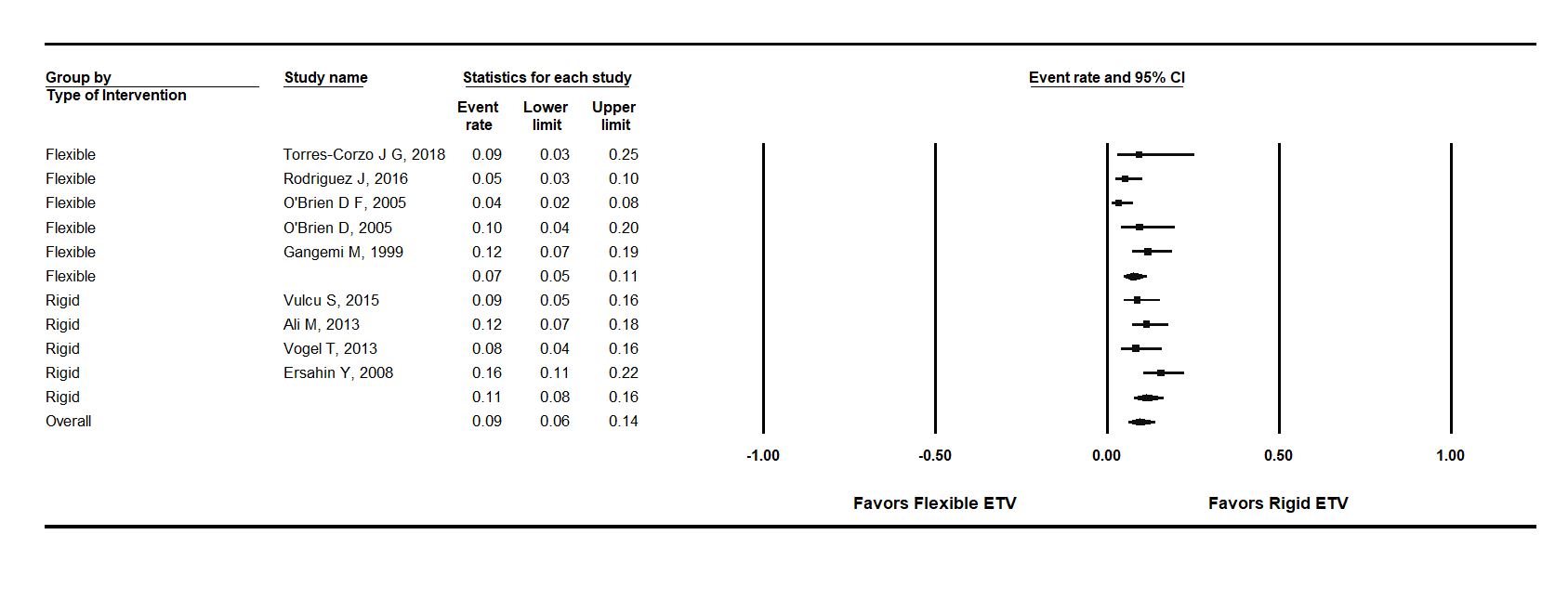


Forest plot for incidence of complication in mixed population stratified by endoscopy type. For flexible ETV: incidence of failure = 8%; number of studies = 5; P-heterogeneity = 0.07; I^2^ = 54%.; for rigid ETV: incidence of failure: 11% number of studies = 4; P-heterogeneity = 0.24.; I^2^ = 27.90%. P-value comparing flexible to rigid = 0.13. Error bars represent the 95% CI. ETV: endoscopic third-ventriculostomy

**Appendix 3. Forest plots for incidence of bleeding**

**Adult population**


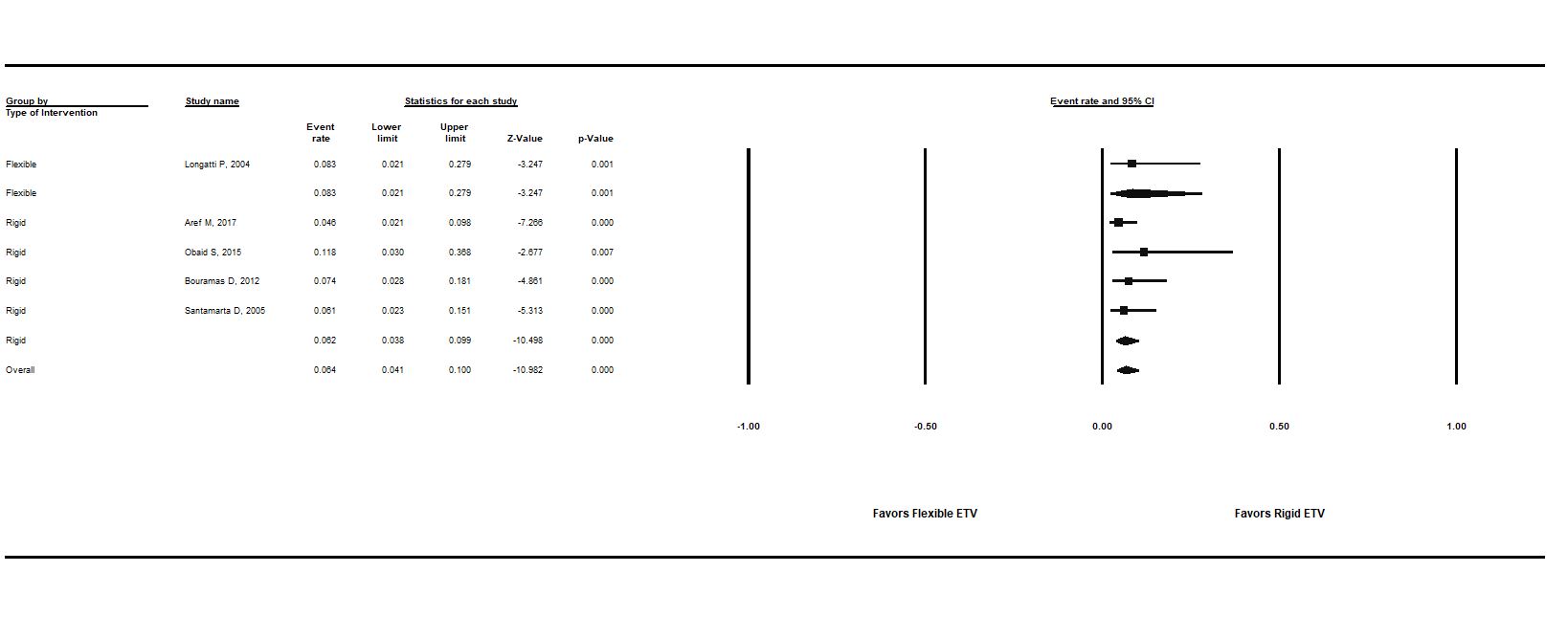


Forest plot for incidence of bleeding in adults stratified by endoscopy type. For flexible ETV: incidence of failure = 8%; number of studies = 1; P-heterogeneity = 1.00; I^2^ = N.A.; for rigid ETV: incidence of failure: 6% number of studies = 4; P-heterogeneity = 0.661.; I^2^ = 0.00%. P-value comparing flexible to rigid = 0.69. Error bars represent the 95% CI. ETV: endoscopic third-ventriculostomy

**Mixed population**


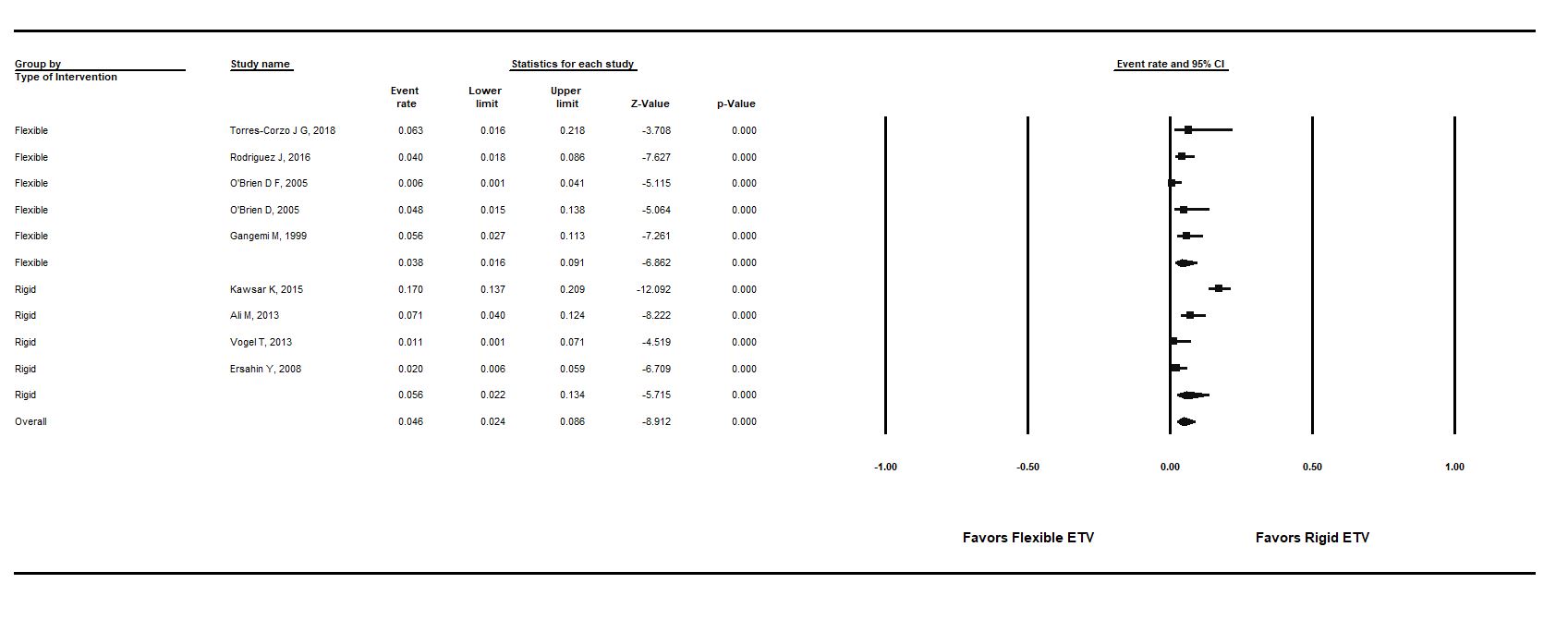


Forest plot for incidence of bleeding in mixed population stratified by endoscopy type. For flexible ETV: incidence of failure = 4%; number of studies = 5; P-heterogeneity = 0.29; I^2^ = 19.1%; for rigid ETV: incidence of failure: 6% number of studies = 4; P-heterogeneity = 0.00.; I^2^ = 89.7%. P-value comparing flexible to rigid = 0.57. Error bars represent the 95% CI. ETV: endoscopic third-ventriculostomy

**Appendix 4. Forest plots for incidence of death**

**Adult population**


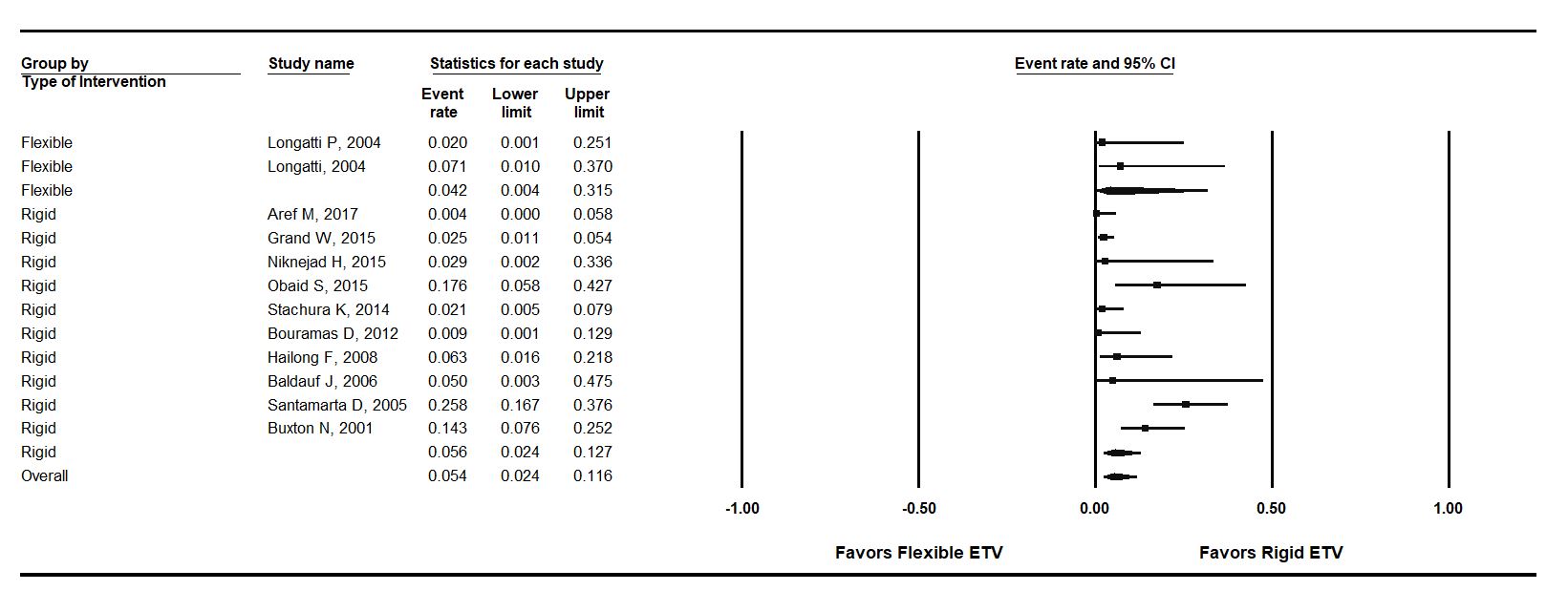


Forest plot for incidence of death in adults stratified by endoscopy type. For flexible ETV: incidence of failure = 4%; number of studies = 2; P-heterogeneity = 0.45; I^2^ = 0.00%.; for rigid ETV: incidence of failure: 6% number of studies = 10; P-heterogeneity = 0.00; I^2^ = 80.90%. P-value comparing flexible to rigid = 0.82. Error bars represent the 95% CI. ETV: endoscopic third-ventriculostomy

**Pediatric population**


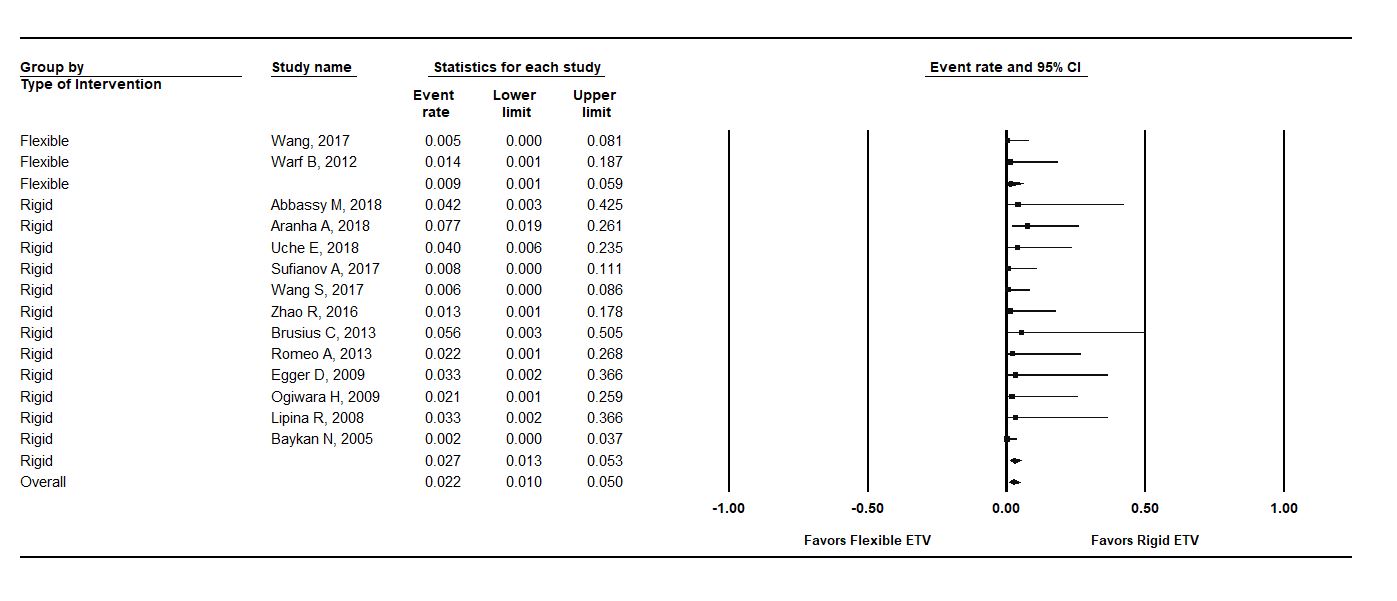


Forest plot for incidence of death in pediatric population stratified by endoscopy type. For flexible ETV: incidence of failure = 1%; number of studies = 2; P-heterogeneity = 0.64; I^2^ = 0.00%.; for rigid ETV: incidence of failure: 3% number of studies = 12; P-heterogeneity = 0.70; I^2^ = 0.00%. P-value comparing flexible to rigid = 0.28. Error bars represent the 95% CI. ETV: endoscopic third-ventriculostomy

**Mixed population**


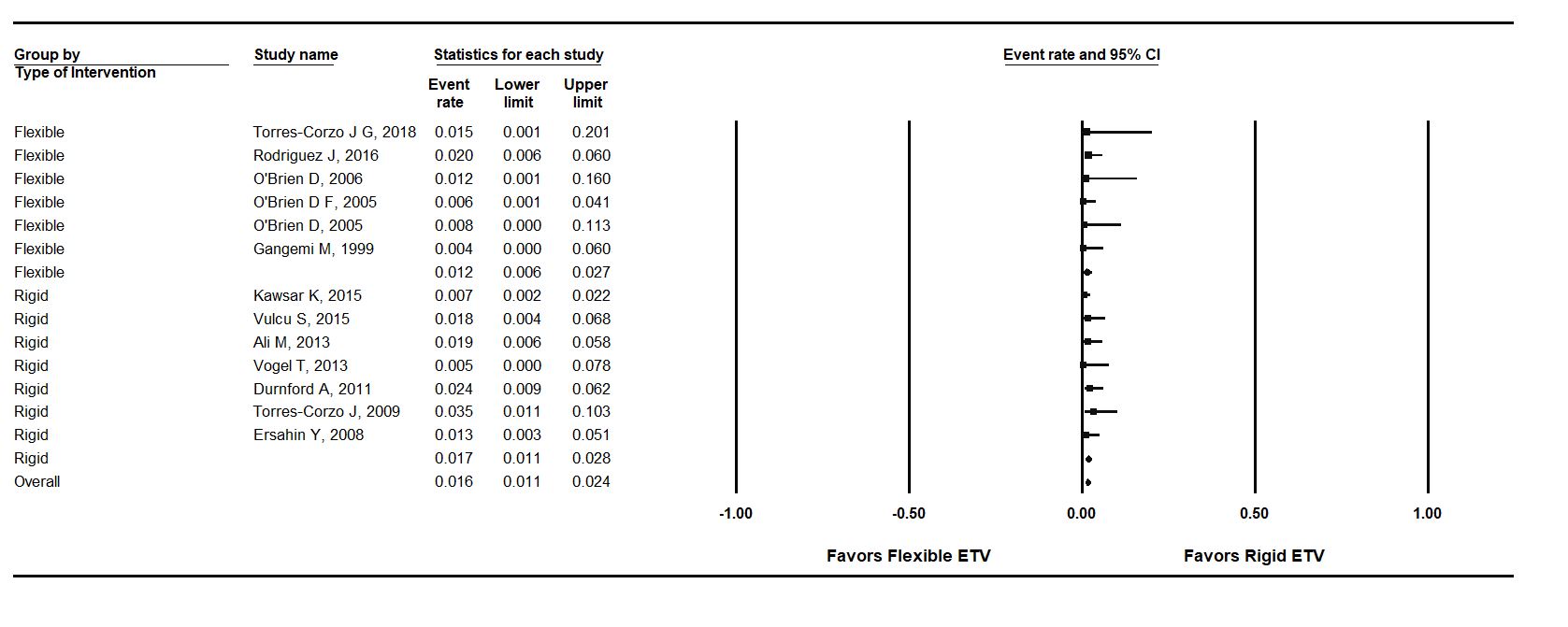


Forest plot for incidence of death in mixed population stratified by endoscopy type. For flexible ETV: incidence of failure = 1.2%; number of studies = 6; P-heterogeneity = 0.84; I^2^ = 0.00%.; for rigid ETV: incidence of failure: 1.7% number of studies = 7; P-heterogeneity = 0.52; I^2^ = 0.00%. P-value comparing flexible to rigid = 0.46. Error bars represent the 95% CI. ETV: endoscopic third-ventriculostomy

**Appendix 5. Quality score assessment**

| **Author, Year** | **Clear Study Objectives/Question** | **Well Defiend Study Protocol** | **Explicit Inclusion/Exclusion Criteria for Participants** | **Specified Time Interval for Patient Recruiotment** | **Consecutive Patient Enrollement** | **Clinically Relevant Outcomes** | **Prospective Outcome Data Collection** | **High Follow-Up Rate** | **Total** |
| --- | --- | --- | --- | --- | --- | --- | --- | --- | --- |
| Abbassy M, 2018 | 1 | 0 | 1 | 0 | 0 | 1 | 0 | 0 | 3 |
| Aranha A, 2018 | 1 | 1 | 1 | 0 | 0 | 1 | 0 | 0 | 4 |
| Chiba K, 2018 | 1 | 0 | 1 | 1 | 1 | 1 | 0 | 0 | 5 |
| Torres-Corzo J, 2018 | 1 | 1 | 1 | 0 | 0 | 1 | 0 | 0 | 4 |
| Uche E, 2018 | 1 | 1 | 1 | 0 | 0 | 1 | 0 | 1 | 5 |
| Wu Y, 2018 | 1 | 1 | 0 | 0 | 1 | 1 | 0 | 1 | 5 |
| Aref M, 2017 | 1 | 1 | 0 | 0 | 1 | 1 | 0 | 0 | 4 |
| Oertel J, 2017 | 1 | 0 | 1 | 0 | 0 | 1 | 0 | 0 | 3 |
| Sufianov A, 2017 | 1 | 0 | 0 | 0 | 0 | 1 | 1 | 1 | 4 |
| Wang, 2017 | 1 | 1 | 1 | 0 | 0 | 1 | 0 | 1 | 5 |
| Rodriguez J, 2016 | 1 | 1 | 1 | 0 | 0 | 1 | 0 | 1 | 5 |
| Zhao R, 2016 | 1 | 0 | 0 | 1 | 1 | 1 | 0 | 0 | 4 |
| Grand W, 2015 | 1 | 0 | 0 | 1 | 0 | 1 | 0 | 1 | 4 |
| Kawsar K, 2015 | 1 | 1 | 0 | 1 | 0 | 1 | 0 | 0 | 4 |
| Niknejad H, 2015 | 0 | 1 | 0 | 1 | 1 | 1 | 0 | 1 | 5 |
| Obaid S, 2015 | 1 | 1 | 0 | 1 | 0 | 1 | 0 | 1 | 5 |
| Vulcu S, 2015 | 1 | 0 | 0 | 1 | 1 | 1 | 0 | 0 | 4 |
| Bisht A, 2014 | 1 | 0 | 1 | 1 | 1 | 1 | 0 | 1 | 6 |
| Salvador S, 2014 | 1 | 1 | 0 | 1 | 1 | 1 | 0 | 1 | 6 |
| Stachura K, 2014 | 1 | 0 | 0 | 0 | 0 | 1 | 0 | 0 | 2 |
| Ali M, 2013 | 1 | 0 | 1 | 1 | 1 | 1 | 0 | 0 | 5 |
| Brusius C, 2013 | 1 | 0 | 1 | 1 | 1 | 1 | 1 | 1 | 7 |
| Melot A, 2013 | 1 | 1 | 1 | 0 | 0 | 1 | 0 | 0 | 4 |
| Romeo A, 2013 | 1 | 0 | 0 | 0 | 0 | 1 | 0 | 1 | 3 |
| Vogel T, 2013 | 1 | 0 | 0 | 1 | 0 | 1 | 0 | 1 | 4 |
| Bouramas D, 2012 | 1 | 1 | 0 | 0 | 1 | 1 | 0 | 1 | 5 |
| Warf B, 2012 | 1 | 1 | 0 | 0 | 1 | 1 | 0 | 0 | 4 |
| Durnford A, 2011 | 1 | 1 | 0 | 0 | 0 | 1 | 0 | 1 | 4 |
| Egger D, 2009 | 1 | 0 | 0 | 1 | 1 | 1 | 0 | 1 | 5 |
| Oertel J, 2009 | 1 | 1 | 1 | 0 | 1 | 1 | 1 | 0 | 6 |
| Ogiwara H, 2009 | 1 | 0 | 0 | 0 | 1 | 1 | 0 | 1 | 4 |
| Torres-Corzo J, 2009 | 1 | 0 | 1 | 0 | 0 | 1 | 0 | 1 | 4 |
| Ersahin Y, 2008 | 1 | 1 | 1 | 0 | 0 | 1 | 0 | 1 | 5 |
| Hailong F, 2008 | 1 | 0 | 0 | 0 | 0 | 1 | 0 | 1 | 3 |
| Lipina R, 2008 | 1 | 1 | 0 | 1 | 0 | 1 | 0 | 1 | 5 |
| Baldauf J, 2007 | 1 | 0 | 0 | 0 | 0 | 1 | 0 | 1 | 3 |
| Idowu O, 2007 | 1 | 1 | 0 | 0 | 0 | 1 | 1 | 0 | 4 |
| Baldauf J, 2006 | 1 | 0 | 0 | 1 | 0 | 1 | 0 | 1 | 4 |
| O'Brien D, 2006 | 1 | 0 | 0 | 0 | 0 | 1 | 0 | 1 | 3 |
| Baykan N, 2005 | 1 | 0 | 1 | 1 | 1 | 1 | 0 | 0 | 5 |
| O'Brien D F, 2005 | 1 | 0 | 0 | 0 | 1 | 1 | 0 | 1 | 4 |
| Santamarta D, 2005 | 1 | 1 | 0 | 0 | 1 | 1 | 0 | 1 | 5 |
| Longatti P, 2004 | 1 | 1 | 0 | 1 | 1 | 1 | 0 | 0 | 5 |
| Longatti P, 2004 | 1 | 1 | 0 | 1 | 1 | 1 | 0 | 0 | 5 |
| Buxton N, 2001 | 1 | 1 | 0 | 1 | 1 | 1 | 1 | 1 | 7 |
| Gangemi M,1999 | 1 | 1 | 0 | 1 | 1 | 1 | 0 | 1 | 6 |

**Appendix 6 Sensitivity analysis for efficacy/failure**

Sensitivity analysis for efficacy/failure outcomes (only includes studies with quality >= 4)

|  |  | **Flexible ETV** | | **Rigid ETV** | |
| --- | --- | --- | --- | --- | --- |
| **Outcome** | **Population**  **type;** | **Effect size**  **(95% C.I.)** | **# of**  **studies** | **Pooled incidence (95% C.I.)** | **# of**  **studies** |
| Failure | Pediatric | 35%  (11%, 70%) | 2 | 29% (18%, 42%) | 15 |
|  | Adult | 50%  (18%, 82%) | 2 | 18% (13%, 25%) | 13 |
|  | Mixed | 21% (14%, 30%) | 6 | 21% (15%, 28%) | 8 |

C.I.: Confidence interval

**Appendix 7 Sensitivity analysis for complications and death**

Sensitivity analysis for safety outcomes of complications and death (only includes studies with quality >= 4)

|  |  | **Flexible ETV** | | **Rigid ETV** | |
| --- | --- | --- | --- | --- | --- |
| **Outcome** | **Population;** | **Pooled incidence (95% C.I.)** | **# of**  **studies** | **Pooled incidence (95% C.I.)** | **# of**  **studies** |
| Complications | Pediatric | 2 %  (0.1%-34%) | 1 | 19 % (7%-46%) | 6 |
|  | Adult | 12 % (3%-40%) | 1 | 8%  (5%-13%) | 7 |
| Death | Pediatric | 1 % (0.1%- 3%) | 2 | 3 % (1%-5%) | 10 |
|  | Adult | 4 % (0.4%- 32%) | 2 | 6 % (2%-16%) | 8 |
|  | Mixed | 1.2 % (0.6%-2.7%) | 5 | 1.7 % (1.1%- 2.8%) | 7 |

C.I.: Confidence interval
